# MAOA promoter methylation and susceptibility to carotid atherosclerosis: role of familial factors in a monozygotic twin sample

**DOI:** 10.1186/1471-2350-13-100

**Published:** 2012-11-02

**Authors:** Jinying Zhao, Christopher W Forsberg, Jack Goldberg, Nicholas L Smith, Viola Vaccarino

**Affiliations:** 1 Department of Epidemiology, School of Public Health and Tropical Medicine, Tulane University, New Orleans, LA, USA; 2 Seattle Epidemiologic Research & Information Center, Veterans Affairs Office of Research & Development, Seattle, WA, USA; 3 Department of Epidemiology, University of Washington, Seattle, WA, USA; 4 Department of Epidemiology, Emory University School of Public Health, Atlanta, GA, USA

**Keywords:** DNA methylation, MAOA, Carotid atherosclerosis, Monozygotic twins, Familial factors

## Abstract

**Background:**

Atherosclerosis is a complex process involving both genetic and epigenetic factors. The monoamine oxidase A (*MAOA*) gene regulates the metabolism of key neurotransmitters and has been associated with cardiovascular risk factors. This study investigates whether MAOA promoter methylation is associated with atherosclerosis, and whether this association is confounded by familial factors in a monozygotic (MZ) twin sample.

**Methods:**

We studied 84 monozygotic (MZ) twin pairs drawn from the Vietnam Era Twin Registry. Carotid intima-media thickness (IMT) was measured by ultrasound. DNA methylation in the *MAOA* promoter region was quantified by bisulfite pyrosequencing using genomic DNA isolated from peripheral blood leukocytes. The association between DNA methylation and IMT was first examined by generalized estimating equation, followed by matched pair analyses to determine whether the association was confounded by familial factors.

**Results:**

When twins were analyzed as individuals, increased methylation level was associated with decreased IMT at four of the seven studied CpG sites. However, this association substantially reduced in the matched pair analyses. Further adjustment for MAOA genotype also considerably attenuated this association.

**Conclusions:**

The association between *MAOA* promoter methylation and carotid IMT is largely explained by familial factors shared by the twins. Because twins reared together share early life experience, which may leave a long-lasting epigenetic mark, aberrant MAOA methylation may represent an early biomarker for unhealthy familial environment. Clarification of familial factors associated with DNA methylation and early atherosclerosis will provide important information to uncover clinical correlates of disease.

## Background

Atherosclerosis is a complex process resulting from the interaction between genetic and non-genetic factors. Despite substantial effort, our understanding of atherosclerosis remains incomplete. Epigenetic modifications, especially DNA methylation, represent an attractive molecular mechanism for atherosclerosis because they may be altered in response to environmental exposures and lifestyle interventions [1]. Indeed, studies in both human [2,3] and animals [4,5] have reported associations of DNA methylation variation with subclinical atherosclerosis and atherosclerotic cardiovascular disease (CVD). However, specific genes and the epigenetic pathways underlying atherosclerosis remain largely uncharacterized.

Monoamine oxidase A (MAOA), encoded by the X chromosome, catalyzes the oxidative deamination of biogenic amines, such as serotonin, dopamine and norepinephrine, and plays a critical role in maintaining the metabolic homeostasis of neurotransmitters. Abnormal MAOA activity has been implicated in several neuropsychiatric disorders [6], and recently pancreatic beta cell function [7] and glucose metabolism [8]. A variable number of tandem repeats (VNTR) of a 30-bp sequence located approximately 1.2 kb upstream of the coding region [9], termed MAOA-uVNTR, has been associated with psychiatric/behavioral disorders [10,11] and cardiovascular risk factors, including body mass index [12], obesity [13], and lipid levels [14]. However, a recent study failed to detect a relationship between an individual’s brain *MAOA* level and *MAOA* genotype [15], suggesting that there are additional regulatory mechanisms that control the expression of *MAOA* gene.

The purpose of this study is to investigate the association between DNA methylation variation in the MAOA promoter region and carotid atherosclerosis, assessed by common carotid intima-media thickness (IMT), using a monozygotic co-twin control design. Because both epigenetic variation [16] and carotid atherosclerosis [17] are under genetic control, it is critical to take into account any potential shared genetic influences between DNA methylation and atherosclerosis. In addition, it is important to control for early family environment because epigenetic variation is influenced by early life experience, which may leave long-lasting epigenetic marks on the epigenome that will likely affect cardiometabolic risk later in life [18,19]. A monozygotic co-twin control design controls for shared genes and early family environment, thus represents a useful model for epigenetic research of complex traits such as atherosclerosis. As far as we are aware, this is the first study to examine the association between *MAOA* gene methylation and subclinical cardiovascular disease, and the potential impact of familial factors on this association in a well-matched monozygotic twin sample.

## Methods

### Study population

Twins included in this study were drawn from the Vietnam Era Twin (VET) Registry, one of the largest twin registries in the U.S. [20] All twins were male veterans who were born between 1946 and 1956. A total of 307 twin pairs (who were raised in the same household) were recruited by the Emory Twin Studies (ETS), which included two companion studies to investigate the role of psychological, behavioral, and biological risk factors for subclinical cardiovascular disease in twins. The ETS include male-male twin pairs, including 187 monozygotic (MZ) pairs and 120 dizygotic (DZ) pairs, with an inclusion of two samples of twin pairs discordant for major depression or posttraumatic stress disorder (PTSD). The ETS protocol has been described elsewhere [21]. This research was approved by the Emory Institutional Review Board, and all twins signed an informed consent.

The current analysis included 84 monozygotic twin pairs from the ETS. These twin pairs were selected based on the availability of DNA samples and phenotype data for both members of a twin pair. All twins were examined in pairs at the Emory University General Clinical Research Center between 2002 and 2010, where their medical history was updated. All twins were Caucasian. Zygosity information was determined by DNA analysis.

### Risk factor measurements

All measurements were performed in the morning after an overnight fast, and both members of a pair were tested at the same time. A medical history and a physical exam were obtained from all twins. Body mass index (BMI) was calculated by dividing weight in kilograms by the square of height in meters. Cigarette smoking was classified into current smoker (any number of cigarettes) versus never or past smoker. Pack-years of smoking were calculated as the number of packs of cigarettes smoked per day times the number of years smoked. Physical activity was assessed by means of a modified version of the Baecke Questionnaire of Habitual Physical Activity used in the Atherosclerosis Risk in Communities (ARIC) Study [22], a 16-question instrument documenting level of physical activity at work, during sports and non-sports activities. The total physical activity score was used in the analysis. Information on alcohol consumption was collected by asking about the number of alcoholic drinks (beer, wine or liquor) consumed in a typical week. The total amount of alcohol consumption (in grams) per week was estimated based on the following algorithms: 4 oz of wine contains 10.8 g, 12 oz of beer contains 13.2 g, and 1.5 oz of liquor contains 15.1 g of ethanol. Hypertension was defined as systolic blood pressure > 140mmHG or diastolic blood pressure >90mmHg. Diabetes was defined as fasting blood glucose >126 mg/dL (7.0 mmol/L).

### Carotid intima-media thickness (IMT) measurement

Common carotid artery IMT was measured using high resolution B-mode ultrasonography by standard techniques [23]. Briefly, IMT was quantified both on the near and far wall at the distal 1.0 cm of the left and right common carotid arteries proximal to the bifurcation. For each segment, the sonographer used multiple different scanning angles to identify the longitudinal image of IMT showing the maximum IMT. At least 10 pictures for each segment were stored digitally, and measurements were made off-line using semi-automated computerized analytical software (Carotid Tools, MIA Inc., Iowa City, Iowa) by one observer blinded to other twin data. Of the stored images, the one with maximum thickness was selected, and IMT measured, for each segment. Average values of the IMT of each of the four segments (right near and far walls, and left near and far walls) were used as the IMT values for each twin in the analysis (total mean of maximum IMT). In order to minimize error, the same technician did IMT measurements throughout the study, and the same equipment and analytical software was used to measure IMT for all the twin participants. In our lab, the mean absolute difference in IMT measured in 7 subjects in whom 2 carotid artery examinations were performed 3 days apart, was 0.03 (±0.02) mm. The mean difference in 2 successive readings of the same 10 segments of common carotid IMT was 0.02 (±0.02) mm with a Pearson correlation coefficient of 0.93.

### DNA methylation analyses by quantitative bisulfite pyrosequencing

**Figure 1:**
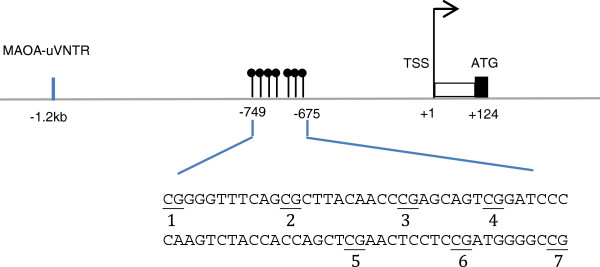
**A schematic illustration for the CpG sites in a promoter region of MAOA gene assayed in this study in relation to the MAOA-uVNTR variant.** The sequence shown represents a 75 bp fragment (−749 to −675 with respect to TSS) in the 5^′^-UTR of MAOA. Numbers 1–7 refer to locations of the CpG sites assayed in this study. TSS: Transcriptional Start Site; ATG: Translational Start Codon.

Genomic DNA was isolated from peripheral blood leukocytes by standard method. DNA methylation level in the MAOA gene promoter region was determined using quantitative bisulfite pyrosequencing by the EpigenDx Inc (Worcester, MA), a company specializing in epigenetic analysis by pyrosequencing. Briefly, the human *MAOA* promoter methylation assay covered seven CpG dinucleotides in the promoter region ranging from −749 base pair (bp) to −675 bp from the transcriptional start site (TSS), based on Ensembl Gene ID ENSG00000189221 and the Transcript ID ENST00000338702. To sequence these selected CpG sites, we designed one pyrosequencing assay and tested for PCR preferential amplification and quantitative pyrosequencing analysis. **Figure**1 schematically illustrates the target CpG sites by pyrosequencing assay with respect to transcription start site (TSS) and the MAOA-uVNTR polymorphism.

The bisulfite conversion was performed with 500 ng genomic DNA using the EZ DNA methylation kit (ZymoResearch, Inc., CA). The PCR reaction was performed with 0.2 μM of each primer with one of the PCR primers being biotinylated to purify the final PCR product using Sepharose beads. The PCR product was bound to Streptavidin Sepharose HP (Amersham Biosciences, Uppsala, Sweden), and the Sepharose beads containing the immobilized PCR product were purified, washed and denatured using 0.2 M NaOH solution and rewashed using the Pyrosequencing Vacuum Prep Tool (Pyrosequencing, Qiagen) as recommended by the manufacturer. Then 0.5 μM Pyrosequencing primer was annealed to the purified single-stranded PCR product. 10 μl of the PCR products were sequenced by Pyrosequencing PSQ96 HS System (Pyrosequencing, Qiagen) following the manufacturer’s instructions (Pyrosequencing, Qiagen). The methylation status of each CpG site was analyzed individually as an artificial T/C SNP using QCpG software (Pyrosequencing, Qiagen). Methylation level at each CpG site was calculated as the percentage of the methylated alleles over the sum of methylated and unmethylated alleles. The mean methylation level was calculated using methylation levels of all measured CpG sites within the targeted region of the gene. Pyrosequencing assay was done on duplicate samples, with a correlation of over 99.8% between the two runs for a same sample. For quality control, each experiment included non-CpG cytosines as internal controls to verify efficient sodium bisulfite DNA conversion. We also included unmethylated and methylated DNAs as controls in each run. In addition, we performed PCR bias testing using Pyrosequencing by mixing the unmethylated DNA control and *in vitro* methylated DNA at different ratios (0, 20%, 40%, up to 100%) followed by bisulfite modification, PCR and pyrosequencing analysis. The percent methylation obtained from the mixing study showed high correlation with expected methylation percentages with a correlation coefficient of 0.97, indicating high quality methylation data.

#### Genotyping of the MAOA-uVNTR variant

To examine whether the association between MAOA methylation variation and IMT is modified by the MAOA-uVNTR, we genotyped the MAOA-uVNTR variant according to the method described previously [24]. In brief, genomic DNA was amplified with forward primer 5^′^-ACAGCCTGACCGTGGAGAAG -3^′^ (fluorescently labeled with FAM) and reverse primer 5^′^- GAACGGACGCTCCATTCGGA -3^′^, with PCR thermal cycling conditions of 10-min denaturation at 95°C, then 35 cycles of 95°C for 30 sec, 60°C for 30 sec and 72°C for 1 min. This was followed by 5-min extension at 72°C. Amplified PCR products were visualized on a capillary-based ABI3100 Genetic Analyzer along with GeneScan 500 ROX as sizing standard. Data collection and allele scoring was performed using GeneScan 3.7 and Genotyper 3.7 (Applied Biosystems). PCR products included 2 repeated sequences in 3 and 4 repeats.

### Statistical analyses

Prior to analysis, continuous variables including IMT and methylation data were logarithmically transformed to improve normality. To adjust for multiple testing, we used the Benjamini-Hochberg false discovery rate (FDR) procedure [25] to correct for the number of CpG sites evaluated and used an FDR-adjusted P value (q value) threshold of 0.05 to determine statistical significance. The PROC MULTTEST procedure in SAS 9.2 was used to calculate the adjusted-FDR (the q-value method).

1) Regression analysis by treating twins as individuals: We examined the association of methylation level at each CpG site and the mean methylation level of all measured CpG sites with carotid IMT, adjusting for age, smoking, BMI, diabetes, HDL, LDL, systolic blood pressure, and physical activity level. These analyses were done using generalized estimating equation (GEE) models, in which carotid IMT was the dependent variable, DNA methylation level was the independent variable, and twin pair was included as a clustering variable to account for the within twin pair correlations.

2) Matched pair analyses by considering twins as members of a twin pair: To examine whether the association between DNA methylation and IMT was explained by shared genetic and/or familial environment, we conducted matched pair analysis by treating twins as members of a twin pair. First, we calculated the intrapair difference in DNA methylation level, defined as the difference in DNA methylation between two members of a twin pair. The intrapair differences in IMT and other continuous variables were similarly calculated. Then we conducted linear regression by regressing the intrapair difference in IMT (dependent variable) on the intrapair difference in methylation level (independent variable) at each CpG site, adjusting for intrapair differences in smoking (pack-year), BMI, HDL, LDL, systolic blood pressure and level of physical activity.

3) Sensitivity analyses: As described before, our sample included twins recruited by two projects with oversampling of twins with major depression or PTSD. To examine whether the oversampling scheme influences our results, we performed separate analyses by further adjusting for depressive symptoms (as measured by Beck Depressive Inventory scores) or PTSD (n = 35 including 9 pairs and 17 singletons). To evaluate the potential impact of MAOA-uVNTR genotype on the association between methylation variation and IMT, we conducted separate analyses by further controlling for this genotype in statistical models. To determine whether combining data from the two studies has an impact on our results, we included study affiliation (THS or SAVEIT) as a covariate in the statistical analyses. In addition, we conducted sensitivity analysis to examine whether batch effects influence our results by including an indicator variable for sample batches (plate 1 vs. plate 2) in the statistical analysis.

## Results

The age of the twins ranged from 48 to 61 years with a mean of 55. Twins included in the current analysis were not different from those not included in terms of IMT and other covariates. Table 1 presents the demographic characteristics of the twins included in this analysis. 

**Table 1:** Demographic, clinical and laboratory characteristics of the twins

**Variable**	**Mean ± SD or %**
Age (years)	55.1 ± 2.8
Type 2 diabetes (%)	11.4
Hypertension (%)	36.5
Current smoking (%)	37.6
Body mass index (kg/m [2])	29.4 ± 4.8
Systolic blood pressure (mmHg)	129.3 ± 17.5
Diastolic blood pressure (mmHg)	81.4 ± 11.6
High density lipoprotein cholesterol (mg/dL)	37.6 ± 10.7
Low density lipoprotein cholesterol (mg/dL)	123.3 ± 36.8
Total triglyceride (mg/dL)	177.5 ± 102.5
Total mean of maximum intima-media thickness (μm)	769.7 ± 121.8
Mean MAOA methylation level (%)	5.0 ± 1.6

The mean methylation level of the seven CpG sites examined in *MAOA* promoter was 5.0%, with the highest and lowest methylation levels occurring at CpG site 5 (7.2%) and site 1 (3.7%), respectively. Methylation levels of the seven CpG sites in *MAOA* promoter were highly correlated with each other (correlation ranges from 0.77 to 0.93, all p’s <0.0001). Carotid IMT was negatively correlated with methylation variation at five of the seven studied CpG sites (Table 2). Mean *MAOA* promoter methylation was also inversely correlated with carotid IMT (correlation = − 0.17, p = 0.03).

**Table 2:** Correlation between carotid IMT and MAOA promoter methylation variation

**Position**	Genomic location^*^	**Methylation level**	**Correlation with IMT**
	**(Relative to TSS, bp)**	**(%, mean ± SD)**	**(P value)**
1	43514718 (−749)	3.70 ± 1.83	−0.17 (0.03)
2	43514729 (−738)	3.73 ± 1.60	−0.22 (0.005)
3	43514740 (−727)	5.44 ± 1.72	−0.10 (0.12)
4	43514748 (−719)	4.34 ± 1.76	−0.20 (0.01)
5	43514773 (−694)	7.24 ± 1.84	−0.16 (0.04)
6	43514782 (−684)	5.61 ± 1.79	−0.15 (0.05)
7	43514791 (−674)	4.67 ± 1.92	−0.12 (0.13)
Mean		4.96 ± 1.65	−0.17 (0.03)

*On chromosome X according to GCRh37/hg19.

1) Results of multivariate GEE by treating twins as individuals: DNA methylation levels at four CpG sites in the MAOA promoter showed significant individual association with IMT after adjusting for known coronary risk factors (all p’s ≤ 0.05). The mean MAOA methylation level was also significantly associated with carotid IMT (p = 0.02). On average, a 10% increase in the mean MAOA methylation level was associated with a decrease of 115 μm (95% CI: 36, 195) in carotid IMT. In the multivariate GEE models, age (p = 0.01) and systolic blood pressure (p = 0.002) were also significantly and positively associated with carotid IMT. Further adjustment for the MAOA-uVNTR genotype attenuated the association between MAOA methylation and IMT. Results for multivariate GEE analyses are shown in Table 3.

**Table 3:** Association between carotid IMT and MAOA methylation variation by multivariate GEE

**Position**	**β**	P^*^	**P**^**‡**^
1	−0.07	0.04	0.07
2	−0.08	0.02	0.03
3	−0.06	0.14	0.20
4	−0.06	0.05	0.08
5	−0.10	0.03	0.05
6	−0.06	0.15	0.23
7	−0.06	0.10	0.14
Mean	−0.09	0.02	0.06

β:regression coefficient; ^*^ P values adjusted for multiple testing and covariates, including age, smoking, BMI, diabetes, LDL, HDL, SBP and physical activity; ^‡^Further adjusting for MAOA genotype.

2) Results by matched pair analyses: DNA methylation levels of the two members within a twin pair were highly correlated at each of the examined CpG site (all p’s < 0.0001). The mean methylation level of the twins within a pair was also significantly correlated (r = 0.38, p = 0.0004). Regression analysis using intrapair differences demonstrated that methylation variation at none of the seven CpG sites was associated with IMT, suggesting that the association observed in individual analysis is largely explained by genetic similarity or other familial factors shared by the twins. Results for matched pair analyses are shown in Table 4.

**Table 4:** Results for matched pair analyses

**Position**	**β**	P^*^
1	−0.002	0.67
2	−0.009	0.10
3	−0.001	0.89
4	−0.007	0.19
5	−0.003	0.42
6	−0.007	0.16
7	−0.006	0.16
Mean	−0.005	0.27

β:regression coefficient; ^*^Adjusted for intrapair differences in smoking (pack-years), BMI, LDL, HDL, blood pressure and physical activity.

3) Results for sensitivity analyses: Additional adjustment for depression or PTSD in matched pair analysis did not affect the association. Further adjustments of the study affiliation (THS or SAVEIT) or the MAOA-uVNTR genotype did not change our results.

## Discussion

We found that DNA methylation variation in the MAOA promoter region is associated with carotid atherosclerosis when twins were treated as individuals, but the association substantially attenuated when twins were analyzed in pairs, a statistical method that effectively controls for genetic background (which is identical in MZ twins) and other familial factors. These results suggest that genetic predisposition and/or shared family environment could confound the relationship between MAOA promoter methylation and carotid atherosclerosis, or represent important antecedents to this association.

There are several ways through which familial factors may affect the relationship between DNA methylation and early atherosclerosis. First, there could be familial confounding factors. For example, children raised in families with low socio-economic status (SES) may have an increased risk of subsequent CVD [26,27], whereas early life socio-economic position is also associated with adult DNA methylation variation [28]. In our study, further adjustments for levels of education and socioeconomic status in adulthood did not affect the relationship between MAOA methylation and IMT. While childhood socioeconomic indicators were not available in our sample, these familial factors were accounted for in the matched pair analyses because twins were raised in the same family during childhood. Because the association between MAOA methylation and IMT substantially reduced after accounting for familial factors, our findings indicate that factors shared by the twins (genetic, parental, maternal, and/or other familial environment) may be important in the link between MAOA methylation variation and atherosclerosis. Second, familial factors may be necessary antecedents to epigenetic alterations or subclinical atherosclerosis [29,30]. Because monozygotic twins are matched on genetic and/or early life family environment, our pairwise analysis effectively controls for these factors.

Recent studies in monozygotic twins have reported associations between DNA methylation variation and human diseases, such as type 1 diabetes [31], psychotic disorders [32,33] and systemic lupus erythematosus [34], indicating that epigenetic variability may contribute to phenotypic discordance between genetically identical individuals [35,36]. Our group also reported associations between global DNA methylation and insulin resistance [37], and between serotonin transporter gene (SLC6A4) promoter methylation and obesity measures [38] in this same monozygotic twin sample. The familial confounding identified in this study, however, does not contradict previous findings. First, monozygotic twins may not be exactly identical [39] and stochastic origins of DNA methylation variation are possible in MZ twins. The two members of a monozygotic pair may harbor subtle genetic differences, such as copy number variation [39], that are associated with an increased level of epigenetic stochasticity. Indeed, a recent large-scale DNA methylation profiling in twins indicates that stochastic epigenetic variation may be more common than we previously appreciated [40]. Second, environment exposed to the two identical twins may not be exactly the same, and environment unique to each member, e.g., disparity in placenta blood supply, may induce epigenetic variation that could potentially increase disease risk. Third, the familial confounding found in this study may represent a disease- and/or gene-specific phenomenon, but not representing the case of other diseases or genes.

In this study, we observed that DNA methylation at particular CpG sites showed considerable variability within monozygotic twin pairs (intrapair difference ranges from −0.08 to −0.24). This agrees with findings by other investigators [31,32]. In addition, the magnitude of DNA methylation variation is relatively small compared to DNA methylation alterations generally observed in cancer. However, the magnitude of methylation variation in our study is comparable to many of the previous studies on nonmalignant complex disorders [31,34,38,40-44]. It is possible that epigenetic variation at multiple CpG sites may be involved in the pathogenesis of complex disease, but each individually confers only a small risk effect to disease. This observation most likely reflects the norm for most human complex disorders and parallels with findings from genome-wide association studies in which many genetic variants contribute to disease risk but the predicted risk associated with each variant is generally small.

A previous study has shown that smoking is closely related to MAOA activity and may also influence *MAOA* promoter methylation [45]. In this study, however, we did not find a relationship between smoking status and methylation level at any of the examined CpG sites. This may reflect a site-specific epigenetic effect on smoking because the two studies examined different CpG sites in the MAOA promoter region (~39kb apart). The lack of association between MAOA methylation and smoking may also be due to low statistical power of our analysis. However, to our best knowledge, the use of 84 monozygotic twin pairs in our study represents one of the largest epigenetic twin studies performed for any complex disease phenotype to date.

Our study has a number of limitations that should be considered when interpreting the data presented here. First, because of practical difficulties in obtaining coronary artery tissues from living individuals, methylation levels were tested in peripheral blood leukocytes, but not directly from arteries or atherosclerotic tissues. Therefore, our results may not provide a direct index of methylation in the vascular system. In addition, our epigenetic data were collected from DNA derived from whole blood leukocytes, which includes a heterogeneous mixture of cell types; as such, we were unable to assess methylation status specific to blood cells. Second, our sample included twins with oversampling of either major depression or PTSD, both of which may influence CVD risk. However, the observed associations between aberrant DNA methylation and subclinical CVD are unlikely to be confounded by depression or PTSD because further adjustment for these two psychiatric conditions did not change the results. Third, DNA methylation influences disease risk through regulating gene expression, which could not be evaluated in our study due to lack of fresh leukocytes or atherosclerotic tissues. Additionally, we could not assess the relationship between DNA methylation and platelets MAOA activity due to lack of platelets samples for the twins. The association of DNA methylation with MAOA gene expression should be investigated in future study. Fourth, we only focused on a small region in the promoter of MAOA gene, whereas epigenetic variation in other genomic regions may also influence disease susceptibility. These regions should be examined in future research. Fifth, our sample size is relatively small which may potentially limit our power in detecting the shared familial factors. However, to the best of our knowledge, a sample size of 84 monozygotic twin pairs represents the largest possible sample size for a matched co-twin control analysis. Finally, our twin sample was derived from a middle-aged sample of male military veterans; therefore, the generalizability to females and other younger or older populations is unknown.

## Conclusions

In summary, in a matched monozygotic twin sample, we found that the association between MAOA promoter methylation and carotid atherosclerosis is largely explained by genetic predisposition and/or family environment shared by the twins. Familial factors, e.g., genetic, parental nutrition, maternal care, and other familial environment, may be a key element that could potentially increase future risk of atherosclerosis through the epigenome. Disentangling epigenetic effects from the confounding influences of genetic and/or familial environmental heterogeneity is critical in elucidating the etiological role of epigenetic variation in disease development and may also provide important information to uncover clinical correlates of disease.

## Competing interest

All authors declared that they have no competing interest.

## Authors’ contributions

JZ conceived the study, conducted the statistical analyses and wrote the manuscript. CWF conducted the statistical analyses. JG, NLS and VV contributed to discussion, interpreted the study findings and revised/edited the manuscript. All authors read and approved the final manuscript.

## Pre-publication history

The pre-publication history for this paper can be accessed here:

http://www.biomedcentral.com/1471-2350/13/100/prepub
